# On Hereditary Insanity

**Published:** 1848-04-01

**Authors:** Rud. Leubuscher


### Original Communications.

### REMARKS ON THE HEREDITARY TRANSMISSION OF INSANITY.

BY DR. RUD. LEUBUSCHER.

THE full and compendious statistical investigations of Baillarger, (Annales Medico-Psychologiques, tom. III.) have again directed the attention of psychologists to the hereditary transmission of insanity. The results deduced by Baillarger, from 600 observations, are as follow:—

1. Insanity on the side of the mother is of more importance, in an hereditary point of view, than on that of the father, owing to its being more frequently transmitted to the offspring generally, and often likewise to several children of the same parent.

2. Insanity on the side of the mother is most frequently inherited by daughters, and on that of the father, by sons.

Baillarger's further conclusions, that the moral capacities of the mother are more frequently transmitted to the offspring, touch upon points scarcely embraced by the limits of the subject, and cannot, therefore, challenge an equal attention with his first-named propositions; although we are not insensible of the attractions yielded by the pursuit of considerations leading more or less far into the domain of hypotheses.

Investigations into the causes of mental diseases will probably always form the leading principle for the development of new theories in psychology. The labour attendant upon a continual accumulation of facts may, however, be greatly assisted by an occasional consideration of general questions, bearing upon the observations already made; for the mind, when it becomes thoroughly imbued, as it were, with the mass of the facts presented to it, is urged towards the investigation of causes; and pausing upon *one* point in the history of development, converts that into a resting place, from whence it may look back upon the way that is passed, and direct its glance forward to the discovery of facts hitherto unobserved.

Two different and opposite hypotheses on mental disease cannot be fairly contested, unless we are able in the case of both to trace them back to the fundamental views on which the causes of the mental disease are based, and without which every attempt at an explanation is inadmissible, although it may be founded on the observation of symptoms attested to by experience. It is in this view of the question that I venture to approach a general, although obscure subject; deeming that the indirect utility of such considerations lies in the admission of an obscurity of which we too often lose sight in the recognition of its general nature. The mind gradually ceases to see a dark object in its true colours, when presented to its consideration associated with other objects equally dark.

Amongst all the causes adduced for mental diseases, there is none more stable and undoubted than hereditary transmission: for as soon as the hereditary character of insanity is admitted, all further discussion is at an end, and the subject becomes exclusively and fundamentally a question of science. Hereditary transmission is also a bye-word of the *Somatic* school. It is an undoubtedly sound proposition, that insanity depends directly upon an actual disturbance of the organization: first, because it may be born with an individual; and, secondly, because it does not proceed from the subjective mental development, or the independent characteristics of the individual, but exists as a part of his being from his very origin. Here the question arises, however, does the simple idea of hereditary transmission explain the complicated process of insanity? or, in other words, is hereditary transmission already a simple idea, incapable of further analysis, when considered from such a point of view as the present state of science admits of our assuming?

It is a fact continually attested by observation, that many insane persons descend from insane parents, and that their children again are insane, and that mental disease may continue and appear through many generations, until it becomes gradually obliterated by the crossing of families in marriage, and is finally extinguished. Does hereditary transmission constitute a perfect and complete product? If this be granted, hereditary insanity must be already determined in its form without development, having a primordially imparted existence. But this is as little the fact as in the case with other diseases. Hereditary tuberculosis passes through individual stages of development no less than the disease in its acquired form: thus, it may have existed in the father as pulmonary tuberculosis, and exhibit itself as tuberculosis of the mesenteric glands in the children. In like manner, a father affected by mania may have a son in whom mental disease shows itself especially as melancholia, and conversely. Hereditary insanity progresses, therefore, through its regular development, in the same manner as the acquired form of the disease, being perfectly *individual* in its character. It assumes a different expression and form, in accordance with the more or less extended sphere presented to it, and likewise in obedience with the variously-manifested corporeal and mental development of the individual in whom it shows itself. If this be granted, the admission involves the necessity of separating each individual case into its elementary causes; and hereditary insanity is then no longer a persistent fact, but is ever changing, and in a continued state of development, like every form of disease. We may admit the peculiar individual mode of development manifested in hereditary insanity generally, and yet be wholly in error as to its actual nature. We may, for instance, suppose that in the form of immediate transmission a certain acridity in the blood of the mother, exercising a direct influence on the fœtus, may constitute the actual essence or being of insanity, and that insanity may thus germinate like a grafted bud in the new individual, somewhat, perhaps, in the manner of the microcosmus of a contagium. It is extremely difficult to arrive here at a clearly-defined conception, when we have no grounds to flatter ourselves with the belief, that our knowledge of the *method* is likewise able to throw light on the *cause*, or, in other words, that an acquaintance with the *genetic process* is also a discovery of the *genesis*. The question of the hereditary transmission of insanity leads us to that of the hereditary transmission of diseases generally—of procreation and conception, of the manner in which we must suppose the origin and independent formation of a third individual to be owing to the combination of two separate individuals, and, lastly, of the dependence in which this third individual is placed with respect to his progenitors. The simple question thus expands into a very comprehensive subject of consideration, and the timid cautiousness with which most writers endeavour to evade the inquiry is no doubt owing to the fear of getting beyond their depth in the great sea of conjecture opened before them. Having made the acknowledgment that these questions will occasionally force themselves upon our notice, I shall endeavour to circumscribe the limits of the subject as much as is compatible with a brief notice of its general bearings.

The offspring or fruit of man and the mammalia is a product emanating from the combined action of two separate individuals—a male and a female. Recent physiology has shown that the direct action of the semen of the male is necessary for the fructification of the ovum, a view in opposition to the older hypothesis, that the semen in a certain manner exercised merely a vivifying influence upon the female organism or on the uterus, and that on the latter depended the further development of the fœtus, as Harvey (*de conceptione*) attempted to prove, in his celebrated comparison of the action of the uterus with that of the brain. We cannot here enter into the consideration of the different hypotheses, whether a seminal animalcule is actually the basis of a new fœtus, (a view which does not allow of being refuted in spite of the ridicule with which it has been met,*) or whether the action of the semen consists in the penetration of the fluid through the capsule of the ovum; the former hypothesis would, however, afford considerable aid in the explanation of hereditary transmission. The fructified ovum is an independent being; it is neither the father nor the mother, nor yet an hermaphrodite, but something intermediate between the male and the female parent; it continues to be further developed through an individual organic force termed a vital force, and designated by Harvey as the *anima vegetiva,* and by Stahl as the *soul.* Its constituent matter is in part inherent, and in part derived, during its development, from the organism of the mother; within which it is formed, and from which it imbibes nourishment.

Considered from this point of view, it is, notwithstanding its individual being, dependent upon the maternal organism, and has the conditions of its existence affected by alterations occurring in the former. This is only made manifest to us in rough outline, and is perhaps most clearly seen in changes of mechanical relations; whilst we are completely

* Bischoff, Entwickelungsgeschichte der Säugethiere und des Menschen. 1842, p. 26. (History of the Development of Man and the Mammalia.) When we consider the extreme minuteness of *one* seminal animalcule, it is easy to suppose that it may be overlooked in the ovum of those animals which deposit the largest ova. In mammalia, other circumstances combine to render investigations on this subject so difficult, that we cannot here arrive at any absolute conviction. Although I am far from wishing to establish a probability to be a positive fact, I must observe, that I regard it as impossible to convince oneself on the first appearance of the embryo, that it has *not* originated in a seminal animalcule. The quantity of cells and cell-nuclei deposited in the germinal layer renders this determination impossible. One single seminal animalcule could not be detected here, even if a microscope of the requisite power were used.

in the dark as to why and how the fœtal blood acts, which is communicated to it by endosmosis and exosmosis, although the existence of this action is proved by the fact, that the fœtus may suffer from diseases through the influence of the mother, whilst still in the uterus. Two modes of transmission present themselves as possible—that imparted by impregnation, and that acquired during the formative development of the fœtus; both of which would manifest themselves at birth as instances of hereditary transmission. The transmission of the maternal relation to the child presents but few difficulties when considered with reference to hereditary transmission, because the fœtus remains throughout the whole period of its formative development in immediate communion with the mother; but how the momentary, and perhaps simply transient action of the semen of the father can impart a resemblance of face and person, and of mental qualities, and a tendency to similar diseases, which, although often not developed till a late period of life, must be imparted at the moment of procreation, appears to be a wholly insoluble mystery. By the acts of procreation and impregnation, the ovum acquires that which we may designate as its type—that is to say, the predetermined mode of development manifested in the individual, according to its race and species, whilst its prolonged sojourn in the uterus cannot be supposed to exert any further change than what is effected in its subsequent existence by means of nutrition. (A proof of this is afforded by the fact that animals, whose development proceeds wholly without the maternal organism, exhibit the type of their progenitors, and possess the capacity of procreating individuals under analogous relations.) The action of the mother appears, however, to exercise the greater influence, and we are likewise of opinion that she yields the germ, which is merely excited into a state of development by the semen of the male. The impregnated germ is, therefore, a being, the individual law of whose formation is in the first place subjected to the law of the genus to which it belongs. Besides this general type of organic structure, which admits of infinite latitude when considered with reference to individuals—as exemplified, for instance, in the peculiar physiognomy of every one—we have also to consider the individuality of the parents of the individual. This is most strikingly exhibited in malformations acquired by parents; thus we find, for instance, that a soldier who lost an eye in battle, had a cyclopic son, (Stahl;) that another man transmitted to all his children a malformation of the little finger, which had been the result of a wound, (Blumenbach.) Various other examples may be found in Rougemont, on Hereditary Diseases (p. 38,) in Stark's General Pathology, and Burdach's Physiology. This resemblance is shown very plainly in the form of the face and figure; and we explain it in the broadest sense by supposing a similarity in the constitution, comprising the form and composition of individual organs, (with the condition of their forces and their sensibility,) or of the whole organism, and a resemblance in mental characteristics manifested in the habits, temperament, talents, and character generally. In the midst of these apparent proofs of the dependence of the new individual, we may still perceive his independence; for although such resemblances exhibit themselves, they do not always do so. They may be fully perfect at birth, as we see exhibited in resemblances of form, (as, for instance, in moles, or in malformations of various parts of the body,) but they may also be gradually developed,* and in that case they undoubtedly require that certain external relations should be present, together with the law to which they are subjected. It is only by degrees that the undefined features of the child acquire a resemblance to those of the father or mother. In some cases we obtain a sort of approximative evidence, as to which of the parents has given the prototype of one organ, or even of the whole organism, but it seems impossible to establish any definite rules on this point. It may be said that the sexes indicate a tendency to follow the same type, the daughters showing more resemblance to the mother in their form, and the sons to the father; an indication of this kind presents itself, for instance, in the accumulation of fat on the buttocks of the Hottentot women, in the structure of the lips, &c., but these cases are simply indicative, being met, on the other hand, by numerous and frequent exceptions. Resemblances in the constitution, composition, and susceptibility of the organism are more complicated in their nature, since we are not even able to indicate with any degree of accuracy the individual causes from which such results emanate. These resemblances are exhibited in the acuteness of certain senses, in the capacity of resisting external influences, (strong parents, for instance, have strong children,) in unusual conditions of the blood—a remarkable illustration of which is furnished by hæmorrhaphilia—in general feebleness, and in the disposition to various diseases. We must remark here, with respect to the character of those diseases that may correctly be termed hereditary, that they frequently do not occur until advanced periods of life. Tuberculosis occurs most frequently in children during the period of growth, the exciting cause of the disease being the tendency to congestion of the lungs, which is so strongly characteristic of early life. But here the questions arise—what had been the state of the disease up to that period? and how did it enter the system? It could not have been present in the form of tuberculosis, but may it not have existed as a minute part of a tubercle, or as that dyscrasic condition of the blood which occasions tubercles? Has the contagion in the blood permeated through the villi of the placenta, or did it already exist in the ovum, or in the seminal fluid? The fact of such a condition as hæmorrhaphilia forbids us to reject the possibility of an abnormal condition of the blood being transmitted, if we would not seek the cause in certain conditions of the vessels. Henle correctly observes, in his "Rational Pathology," that we cannot arrive at certain ideas regarding hereditary transmission, owing to our ignorance of the first manifestations of the pathological process, as well as of the inherent affinity existing between certain forms of disease, although we may have some intuitive notion of the connexion existing between them; as, for instance, between tubercles and scrofula, gout and hemorrhoids, &c. I have thrown in all the above questions, with a view of showing how undefined and confused are our ideas of the dispositions

* Gaubius relates an instance of a man who had the little finger bent into the hollow of the hand, and in whose sons the same peculiarity made its appearance at the same age.

affecting even corporeal conditions, and yet these furnish us with the most stable point for the security of our views regarding the mode of action, and the accidental cause of each individual case.

Every-day experience testifies to the transmission of mental resemblances. We may certainly, in many cases, ascribe these to education, and to the influences at work around the child, by which the mind is led in the same direction, until a character which has been originally acquired from the love of imitation, becomes, by continual repetition, second nature. But apart from such influences, we see the whole mental direction of the parents reflected in the most surprising manner in their offspring; thus, we often observe, even in children who have been separated from infancy from their parents, that there exists in both the most striking similarity in carriage, deportment, way of moving, in the proficiency evinced in certain pursuits, and likewise in the higher stages of mental development, as in the mode of thinking and reasoning. Here, again, we have a general indication, modified to a certain degree by sex, exemplified in the case of sons, who often inherit, together with the corporeal habitus of the mother, a greater softness of disposition. The hypothesis that maintains the existence of a soul separate from the body, must explain the hereditary transmission of mental characteristics by the solution of two mysteries of creation—a similar body and a similar soul. To me it appears that hereditary transmission is a fact in opposition to such views, and cannot be disregarded in the discussion of the organic conditions of the soul. Whatever idea we form to ourselves, whether we regard the soul as the force of matter, as a substance, or a monas, it must still have been elaborated from the fulness of the whole perceptive world, before it could give expression to the higher mental activity of reason and free-will; and even in this exalted sphere of contemplation, the world of the senses is ever blending with the higher psychical world.

The development of psychical activity must vary according to the difference existing in the organs, and the more points of contact the latter present with the external world, the greater will be the mass of matter exposed to their action. Thus, wherever certain organs possess a peculiar constitution, which although we are not able always to indicate in individual cases, we can perceive in a general form, it gives a corresponding colour to the psychical qualities. If we suppose the germ to have a disposition to resemble the parents, and that the psychical qualities are developed through it, this reflection of psychical phenomena no longer strikes us as singular. We know from experience that a resemblance, with certain habits of body, frequently goes hand in hand with a resemblance in mental characteristics; and further, as is proved in hereditary transmission of mental qualities, that this resemblance frequently does not manifest itself until subsequent and different periods of life—as, for instance, on the occurrence of puberty, where the possibility of similar actions is imparted with the newly developed capacities of the organs. This resemblance, or hereditary transmission, is, however, in no case perfect, being always modified according to the peculiar conditions of the individual. Thus the hysterical spasm of the mother may be transmitted to the daughter, modified into mere extreme irritability, which requires the occurrence of peculiar circumstances to assume the form of convulsions; in like manner, considering the question psychically, the excessive sentimentality of the mother may show itself in her offspring in an easily excited imagination, or in a peculiarly weak and soft disposition, which may be corrected by sensible guidance, or develop itself, under favouring circumstances, into a similarly sentimental ardour. In the same manner, the transmission of mental characteristics is modified according to the law of individuality.

With these preliminary remarks, we will now proceed with the consideration of insanity. The fact of its hereditary transmission has always been admitted as established, and may be found attested in all statistics on the subject. The data vary, however, very considerably. Thus, Burrows asserts that in six-sevenths of his patients the disease was hereditary—a relation which is, however, by no means confirmed by the investigations of others. Esquirol found 150 cases of hereditary disease among 264 private patients, but only 105 among 466 patients at the Salpetrière. Lautard reckons only one-fifteenth of the cases hereditary, basing his calculations on the data furnished by the Insane Asylum of Marseilles. (See Griesinger, p. 113.) No one has, as far as I know, made higher estimates than Baillarger of the hereditary transmission of insanity. We see, however, on comparing the separate tables, that they err, from the circumscribed nature of the data being limited to one asylum, and often to the results yielded in a period of some few years. Besides this, as Griesinger correctly observes, they are likewise, in a great measure, erroneous, from being drawn up in accordance with different principles; the one case simply taking note of the disease of the parents, or grand-parents of the patient, whilst in another case the disease of collateral relatives is considered, as, indeed, it necessarily ought to be, since the intervention of other causes may have prevented the exhibition of the disease in the immediate relatives, whilst favouring dispositions may develop it in collateral branches of the same family. Many questions of the greatest practical importance here present themselves, which it has been hoped would be most readily and satisfactorily answered by means of statistical tables. Is mental disease transmitted more frequently from the mother than the father? This question, considered in its strictest sense, might show that insanity depends mostly upon conditions which are either limited to the mother or father exclusively; that it is connected with this or that system and organ; or, finally, that the influence of one or the other parent predominated in the act of procreation. We thus see how a question of this nature opens the way to a whole series of others, and how cautiously we ought to proceed in giving a decisive reply, lest we involve ourselves in a mass of hypotheses, in our attempt to explain the subject under consideration. Even Esquirol established the proposition that mental disease was more frequently transmitted from the mother than the father—an opinion that has continued in force, and has been very nearly considered as a settled fact by Baillarger. Another point, tolerably well attested by experience, and scarcely to be refuted on theoretical principles, has again been generally advanced—namely, that insanity is not so frequently transmitted to the offspring when it does not show itself in one or the other parent until after the birth of the former, excepting where the disease is based on hereditary disposition, and appears to have been simply retarded in its manifestation. It would be very important to learn how far it is necessary to extend our investigations amongst the relatives of a patient, and through how many generations the disposition may be transmitted; but these are points into which statistical inquiries do not, and cannot enter.

There is another consideration which presents great difficulties in a practical point of view. Thus a disposition or hereditary tendency to mental diseases is justly regarded as including an hereditary disposition to acute cerebral and nervous diseases, to epilepsy, &c. Griesinger quotes a case mentioned by Rush, of a mechanic who died from the effects of a second attack of madness, and whose six children all suffered from headache, although none of them exhibited any symptoms of derangement. We are here strongly led to consider the headache of the children as a symptom of the same cerebral condition which in the father induced insanity. Epilepsy presents frequent illustrations of this hereditary transmission. Suicide has been ascribed to the same tendency, but in most cases it is nothing more than a symptom of mental disease. There are many curious instances on record where whole families have been swept away by suicide. I will only cite one case mentioned by Esquirol, (vol. i.)—"A rich merchant of an impetuous character, was the father of six children, to each of whom he gave a considerable sum of money when they were all fully grown up. The youngest son became melancholy, and at the age of 26 or 27 threw himself from the top of the house. One of his brothers became very much depressed at his death, and after frequent attempts at suicide, died a year afterwards from often repeated prolonged fastings. The following year another brother had an attack of mania; a fourth, who was a physician, and who two years previously remarked to me, with horrible coolness, *that he should not escape his fate*, killed himself. Two or three years afterwards, a sister was affected by mental disease, and made many attempts to destroy herself; and lastly, the sixth brother, who lived in the happiest domestic relations, and seemed in consequence to have been spared during many years, also committed suicide."

There seems every reason to consider that in some cases of depravity and crime we ought to take into consideration the probable existence of an hereditary tendency, especially where the crime seems to have been merely the result of organic disease. Such cases are, indeed, generally regarded as manifestations of mental disease where they are characterized by favouring conditions; whilst, under other circumstances they fall under the jurisdiction of the law. We may consider insanity as hereditary, where the parents, although not insane, are in a condition commonly expressed as having a screw loose, are distinguished by certain eccentricities of character, by waywardness, and by an inclination to certain passions. There is at present at the Hospital of La Charité, a tradesman who suffers from a deficiency in the power of will.* His father attends to his business with great assiduity, but has, besides other pecu-

* I know of no other terms by which to express the German word *Willenlosigkeit*.—TRANSLATOR'S NOTE.

liarities, the crotchet of going every day, at the same hour, to a certain spot in the city, where he turns himself round several times, exhibiting himself with such punctuality, that his appearance may be determined to the minute. A case like this may certainly be regarded as one of partial derangement. There are others, again, in which, under uniform relations, some unusual peculiarity may be manifested, simply as such, for years and years, until, becoming suddenly connected with a number of other morbid psychical conditions, it forms a ground-work for insanity. How far are we justified in regarding a morbid condition of the nervous system, suicide, crime, and eccentricity on the part of the parents, as the ground of the insanity of their children? Statistical reports can only furnish broad and general views of such relations, for in the manner in which they are drawn up, they do not profess to afford, and, indeed, cannot give any account of particular conditions; but, notwithstanding, they serve as the clue for the solution of the most important practical questions.

The insanity of the children is, moreover, not necessarily hereditary because the father or mother may have been insane, since it may have originated as a primary condition in the children. In order to prove with certainty the hereditary nature of the disease, we require in each individual case an especial proof that the predisposition of the child is similar to that of the parents—an investigation which is only practicable on taking into account all the separate circumstances that may have exercised a special influence in each individual case. Further, in order to be able to assert that insanity is hereditary, we ought, at least, to be able to show that it has originated under conditions identical with, or analogous to, those affecting the parents or relatives. To effect this, it would be necessary to have an intimate acquaintance with the condition of the forefathers of the patients, which is only practicable (even in an approximative degree) by the physician of an insane asylum, who may chance to have presided half his lifetime over a provincial institution of this nature, which must further have constituted the central union for the insane of the whole district.

Another method of inquiry seems to me to admit of practical application, and is based upon an attempt to find in the form, manner, and course in which the hereditary disease has manifested itself, some connecting links to guide us in our judgment. If strictly defined rules are to be sought for in what I simply venture to consider as indications, we might, perhaps, discover from the case itself, without having any knowledge of its anamnesis, whether or not it could be regarded as hereditary.

*The lower forms of mental disease, as imbecility or silliness, and various forms of depression*, appear, to a remarkable degree, to be of an hereditary nature. We must not regard cases of mania as refutations of this fact, since melancholia may be attended with mania, the latter being actually a mere transient condition, an individual stage, more strongly developed than any other in the disease, and therefore the one that appears most conspicuously exhibited, but not on that account to be esteemed as the basis of the whole disease. The character of hereditary forms of insanity appears to be especially depressive in its nature; whilst insanity, when it manifests itself under a form of excitement, is imaginative, and disposed to build up a whole system of fantastic illusions, can only, in very rare cases, be considered as of an hereditary character. The phases of insanity to which we have alluded are characterized by mental activity, and a certain luxuriance of fancy, a degree of vital integrity, which even in the midst of the mental disturbance, shows a glimmering of noble qualities in the very richness of its hallucinations, and in the fulness and diversity of its combinations. In cases of hereditary insanity, on the other hand, the powers of the mental development are primordially interrupted and disturbed. The influence of the external world, the *perceptions,* in the widest sense of the term, are modified by the disposition of any one certain organ, and this variety is impressed upon the organs without, on that account, destroying the independence of consciousness. An attentive consideration of many cases of hereditary insanity has always produced an impression upon my mind, as if a load were weighing down the patient, and preventing his exercising any power either to shake off or develop the insanity with which he was afflicted. In persons gifted with high mental endowments, and still intent upon their own psychical improvement, who do not yield without a struggle to the sway of organic mental disease, this weight appears to manifest its action by inducing extreme depression and sadness.

Cases of hereditary insanity, when not acute, are very frequently of such a nature that there is much difficulty in knowing how to dispose of them—the patients being unfit to remain in their own families, and yet scarcely admissible in an insane asylum, or in any kind of penitentiary. Unconscious of their own condition, and full of whims and oddities, they wander about, vegetating harmlessly on, for the most part, but still requiring careful watching, on account of the fear of occasional outbreaks, which might endanger the safety of others. When such patients come under medical treatment, owing to some acute attack of the malady, they cannot be brought beyond the same uncertain and merely relative degree of amendment.* Acute attacks of this nature are proportionally more speedily cured than in cases of primary insanity; but even when the violence of the symptoms has been moderated, there still remains the same diseased *individuality,* so to speak, whose previously established characteristics give a certain guarantee of a speedy relapse. There still remains the same hallucination of the senses, which must be referred, in such cases, to a diseased state of the sensorial organs, or of the sensorium commune, arising directly from morbid sensations, which occasions impressions of actual circumstances conceived under false ideas, but not originating in the representation conveyed by the imagination to the perceptive faculties. The physician may succeed in explaining to his

* These conditions might perhaps be correctly arranged under the head of *moral insanity;* a term greatly in request in the present day in psychology. *Prichard,* who has paid especial attention to diseases of this nature, states that *moral insanity* is in many cases of hereditary origin, (Treatise on Insanity, p. 12.) I have, however, only cursorily alluded to this expression, as it is an ambiguous term, embracing the most various conditions. I shall, however, elsewhere enter more fully into the consideration of this subject.

patients the fallacy of their conceptions; but the source of the evil within the organism will still remain, baffling all the efforts of medical skill. Attacks of hereditary insanity are likewise characterized by more or less regularly *periodic* intermissions. I believe, however, that with the exception of epilepsy, and some other forms depending upon spasmodic conditions of the nervous system, there is no form of purely periodic insanity, or such as presents perfect intermissions between the separate attacks. Although it may not be possible, in intermittent disease, to discover, during the intervals between the individual paroxysms, which organ is morbidly affected, we are often able to recognise the causes that induce a recurrence of the attack by studying the tone of mind, and the character of the impressions excited in the imagination, notwithstanding the apparent calmness of the disposition during the intermittent periods, and the care with which the patient frequently conceals his passions and desires. We are not yet sufficiently impressed with the conviction, that the origin of insanity must be traced to higher sources than can be detected in the mere paroxysms of mania.

True insanity seldom manifests itself in children; and we find that the hereditary disposition is not developed into insanity before the age of puberty, unless, indeed, it may arise from some deficiency in the brain, in which case it produces imbecility.

There are many very curious cases recorded, in which the disease broke out in the children exactly at the same periods of life in which it had manifested itself in the parents; and, likewise, where it occurred at the same age in various collateral branches of a family. Thus, Esquirol mentions the case of a woman, who, after childbirth, when in her twenty-fifth year, became insane, and whose daughter was also attacked at the same age, and after her confinement. He further relates the following fact, quoted from Rush*:—

"Captains C. L. and J. L. were twins, and so much alike, that they could not be distinguished apart. They served in the American war of independence, distinguished themselves equally, and obtained the same military rank. They were of cheerful dispositions, were happy in their respective marriages, and in the enjoyment of easy means. Captain C. L. was at Greenfield, two miles from the residence of his brother, Captain J. L., who returned from the meeting of the State of Vermont, and killed himself, after having been depressed in spirits and sullen for some days past. At the same time, Captain C. L. became melancholy, and spoke of committing suicide. A few days afterwards, he proposed to his wife one morning to take a walk, and, going into an adjoining apartment, cut his throat. The mother of these two brothers was insane, and two of her sisters were tormented during many years by a desire to commit suicide."

I have myself met with two cases at Halle, where insanity broke out at the same age in which the parents of the patients had been seized. One of these, a peasant, and who had been of weak mind from his childhood, but good-natured, became a maniac in his twentieth year, in consequence of his taking too much to heart an expression made use of by

* I have looked through Rush's collected works, at the Medico-Chirurgical Society, in order to give the case in his own words, but I have been unable to find the passage referred to.—TRANSLATOR.

a preacher, "that all men were sinners." The father had been seized with mania at the same age. The other case was that of a woman, between forty and fifty, who had not yet ceased to menstruate. Her mother had become insane at the same age; a brother was insane, and a cousin on the mother's side, who was likewise in the asylum. She had always lived happily with her husband, had never had any sickness, had healthy children, and the only exciting cause to be found for her insanity was a strife with a neighbour, in which she had made use of the asseveration, "May the devil take me!" After this, she believed she had sinned against the Holy Ghost, became melancholy, made several attempts to commit suicide, and fell into a state that rendered it necessary to place her in an asylum. Nothing more definite regarding the insanity of the parents was to be obtained in either case.

These coincidences in the time of the attacks seem to be most easily explained by the iufluence of the different periods of development; and, as far as I have been able to gather from a comparison of the cases observed, these coinciding attacks agree, especially in women, with one of the periods of sexual development; and, I believe, I may establish the proposition, *that the outbreak of an hereditary disposition to insanity is especially connected with these processes of development*, as the occurrence of puberty, childbirth, and the climacteric period. In men, these processes in the organism are not manifested by external signs; but there can be no question that they exercise the greatest influence on the whole structural activity, both of body and mind. There is at present in La Charité a young girl of twenty, whose brother is also insane. She was first temporarily deranged in her sixteenth year, on the occurrence of menstruation, and was always deranged whenever there was a deficiency in the menstrual discharge, or a delay in its recurrence. She raves about a prince of the blood royal whom she fancies she sees everywhere, and is in a constant state of depression, and absorbed by her hallucinations and dreams. Menstruation has not occurred during the eight months that she has been in La Charité. If the proposition I have advanced be correct, we may regard a case of insanity as hereditary when it appears in the child on the occurrence of puberty, and in the mother at the climacteric period. Coincidences in the ages at which insanity breaks out are not without exceptions. Insanity must, however, necessarily be influenced by the differences characteristic of different periods of life. But it would seem impracticable to define the form more exactly—as, for instance, that on the occurrence of puberty mania prevails, whilst at the climacteric period, cases of melancholia predominate, since a thousand individual circumstances may intervene to render the assumption invalid; and this undefined character—this change in external forms—is a main proof of the modification of hereditary insanity, according to the law of individuality.

It is practically important to inquire into the conditions that maintain the hereditary disposition, and favour an outbreak of insanity. It is, of course, evident that the same relations which occasioned and fostered insanity in the parents, cannot be without influence on the children; amongst these, taken in the broadest sense, we may reckon influences of air and food; and lastly, domestic relations—social standing. Where the necessities of life, and a struggle with daily want, have early affected the mind of a person organically predisposed to insanity, or where he is weighed down by the same adverse pressure that had already impeded the mental activity of the parents, and grows up in the same sphere of prejudices, which sooner or later may bring him into collision with the world, insanity will need but a slight incentive to develop itself. Education exercises a twofold influence. In persons of common-place character, who do not themselves know what they would, or what they should do, education, as has already been observed, is nothing more than blind habit. The susceptible mind of a child acts at first only reproductively, reflecting the impressions which it has received. Even in manhood, we find it difficult to tear ourselves from the fetters of habit; and this is more especially the case, where those surrounding us are persons whom we have learned, from moral considerations, to regard as patterns. The direct instruction imparted to children by a parent labouring under mental disease cannot yield sound fruits. We must here not only take account of manifest insanity, but also of those conditions of mind, which for years together may continue to exist as actual insanity, but which, from the absence of any exciting cause, have not made the patient a fit subject for an insane asylum. By way of illustration, I will here cite a case that occurred at Halle. The son of a weaver, who was himself said to have been a person of peculiar habits, lived with his imbecile mother. Although of very weak understanding, the son had learned to earn his own livelihood, but the mother prevented him from working during the last few years of her life. In the Statistical Report, it is stated that she was too proud to let her son do menial work, as she had once been in better circumstances. The son became completely brutified, and both these wretched imbeciles remained a whole winter shivering with cold and hunger, until at length the son fell into such a state, that he lay continually in the midst of his own excrements, or moved about on all fours, terrifying the inhabitants of the village by his savage howls. When once removed to the asylum, he was speedily brought back to the habits of a human being. But it is a mournful reflection that such things are, and can attain to such a height!

The psychical condition of relatives may exert an influence still in another way. Insanity stands very far removed from ordinary life, and it is best, perhaps, that the generality of men should regard it as something monstrous, and entirely apart from their existence; but when it is brought near to us in the person of some beloved relative, and we recognise its deep-rooted existence in the midst of ordinary life, we are irresistibly led on to a train of self-examination; the deeper our love for the sufferer, the more intimately do we enter into his condition. We see the impurity and irrationality within our own minds, and such considerations may be directly based on a weak and undeveloped self-consciousness. The consciousness that one descends from insane parents, and bears the germ of insanity within one's organism, acts as a powerful incentive to the outbreak of the disease. The fear of an epidemic is the surest means of being attacked by it. Schlegel (in his work on Nostalgia and Suicide, Hildburghausen, 1835) gives, with reference to suicide, some illustrations of the powerful action of such representations, even where there is no hereditary disposition to insanity. A man hanged himself, because in his youth he had stood beside the gallows on which a convict had been executed; he considered the spectacle as a warning of a similar fate to himself, and after being tormented for years by this superstitious dread, at length inflicted on himself the death he had foreboded. Another case was that of a young girl who had been seduced, and who threw herself into the Reuss, near the Devil's Bridge, after having written to her seducer that her ghost would haunt him until he found the same grave as herself. The man at first received the announcement with much indifference, but, by degrees, it made such an impression on his mind, that some years afterwards, being accidentally in Switzerland, he felt himself irresistibly drawn towards the same spot, and sprang into the stream. The above case, which is taken from Esquirol, may be compared with Ideler's article on Suicide, in the Encyclopædia of Medical Sciences by Busch, Dieffenbach, &c., vol. xxxii.

Even a *partial* cure of hereditary insanity can only be effected by a carefully conducted prophylaxis from early childhood. The noble exertions made by Guggenbühl in relation to kretenism, have proved efficacious even where there was deep-seated organic disease of the brain. But we must not flatter ourselves that the malady can always be averted, notwithstanding the most watchful mode of treatment.

By the crossing of the members of different families in marriages, the disposition may be obliterated, as it may likewise be continually strengthened by marriages in the same race and family; certain rules do not, however, appear admissible with respect to the capacity for the transmission of insanity. All that the State can do is to establish a law to prevent *insane persons* from contracting marriages, and thus perpetuating disease.

---

